# Activation of CCL21-GPR174/CCR7 on cardiac fibroblasts underlies myocardial ischemia/reperfusion injury

**DOI:** 10.3389/fgene.2022.946524

**Published:** 2022-09-09

**Authors:** Xiao-Wen Meng, Mian Zhang, Jun-Kai Hu, Xin-Yu Chen, Yu-Qin Long, Hong Liu, Xiao-Mei Feng, Fu-Hai Ji, Ke Peng

**Affiliations:** ^1^ Department of Anesthesiology, First Affiliated Hospital of Soochow University, Suzhou, China; ^2^ Institute of Anesthesiology, Soochow University, Suzhou, China; ^3^ Department of Anesthesiology and Pain Medicine, Davis Health System, University of California, Davis, Sacramento, CA, United States; ^4^ Department of Anesthesiology, University of Utah, Salt Lake City, UT, United States

**Keywords:** myocardial ischemia/reperfusion injury, differentially expressed genes, CCL21, GPR174/CCR7, cardiac fibroblasts, transcriptome

## Abstract

**Background:** The mechanisms underlying myocardial ischemia/reperfusion (I/R) injury are not fully understood. This study aims to explore key candidate genes and potential therapeutic targets for treatment of myocardial I/R injury.

**Methods:** The transcriptional profiles of ventricular myocardium during cardiac arrest, ischemia, and reperfusion were obtained from the Gene Expression Omnibus database. Based on the transcriptional data of GSE6381, functional pathway and process enrichment analyses, protein–protein interaction network, and gene set enrichment analyses were conducted. In the animal experiments, we established the myocardial I/R injury model in mice. We validated the mRNA and protein expression of the key genes using the qPCR and western blots. We further assessed the expression and localization of CCL21 and its receptors using immunofluorescence staining experiments.

**Results:** The microarray analyses identified five key genes (*CCL21*, *XCR1*, *CXCL13*, *EDN1*, and *CASR*). Myocardial I/R process in mice resulted in significant myocardial infraction, histological damage, and myocardial apoptosis. The results of qPCR and western blots showed that the expression of CCL21 and CXCL13 were increased following myocardial I/R injury in mice. Furthermore, the immunofluorescence staining results revealed that the expression of GPR174/CCR7 (CCL21 receptors), but not CXCR5 (CXCL13 receptor), was elevated following myocardial I/R injury. Moreover, the activated CCL21-GPR174/CCR7 signaling was located on the cardiac fibroblasts of the myocardium with I/R injury.

**Conclusion:** This study revealed several key factors underlying myocardial I/R injury. Of these, the activation of CCL21-GPR174/CCR7 signaling on cardiac fibroblasts was highlighted, which provides potential therapeutic targets for cardioprotection.

## Introduction

Timely restoration of blood flow is the most effective therapeutic strategy to salvage the viable myocardium for patients with acute myocardial infarction (Moran et al., 2014). Nevertheless, reperfusion *per se* leads to myocardial ischemia/reperfusion (I/R) injury, a complex pathophysiological process contributing up to 50% of final myocardial infarct size (Boag et al., 2015). Several characteristics of myocardial I/R injury are inflammatory responses, reactive oxygen species accumulation, cell apoptosis, and intracellular calcium overload (Hausenloy and Yellon, 2013; Zhang et al., 2017; Ge et al., 2019; Yuan et al., 2019). Despite recent progresses attempting to attenuate myocardial I/R injury, it remains an unsolved problem.

During cardiac surgery under cardiopulmonary bypass, myocardial I/R injury is predictable and inevitable (Bulluck et al., 2016). Compared with an unprotected myocardial I/R process, blood cardioplegic arrest and protective hypothermia are utilized to limit myocardial injury. A previous study assessed the gene expression profiles of myocardium in the early I/R stages in cardiac surgery using the microarray approach (Arab et al., 2007). Their results suggested that multiple signaling pathways such as PI3K signaling and hemoglobin synthesis were activated during the process (Arab et al., 2007). That study showed gene expression profiles only, without any further analysis or experimental validation. To date, the mechanisms of myocardial I/R injury are not fully understood.

This study aimed to explore the key genes and pathways in myocardial I/R injury. Through analyzing the transcriptional data, differentially expressed genes (DEGs) were identified from the Gene Expression Omnibus (GEO) database, and functional pathway and process enrichment analyses were performed. For the key genes, Gene Ontology (GO), Kyoto Encyclopedia of Genes and Genomes (KEGG), and protein–protein interaction (PPI) were investigated. Gene set enrichment analysis (GSEA) was used to reveal enriched pathways in the stages of ischemia and reperfusion. Finally, the expression of key candidate genes was assessed in myocardial I/R injury of mice, and the activation of CCL21 with its receptors GPR174/CCR7 was highlighted.

## Materials and methods

### Data acquisition and differentially expressed genes identification

The gene expression profile dataset GSE6381 was downloaded from the GEO database (https://www.ncbi.nlm.nih.gov/geo/). GSE6381 presented early gene expression profiles during intraoperative myocardial I/R in cardiac surgery under cardiopulmonary bypass. Specifically, human right ventricular samples were obtained during surgical repair of ventricular septal defect and resection of right ventricular outflow obstruction, with four replicates at each stage (immediately post-cardioplegic arrest, end-ischemia, and 5 min after reperfusion). Data were detected on the Affymetrix Human Genome U133A Array platform (GPL96). The limma package in R language was used to identify DEGs for end-ischemia vs. cardiac arrest, reperfusion vs. end-ischemia, and reperfusion vs. cardiac arrest, respectively (Ritchie et al., 2015). The thresholds for DEGs selection were set as *p* < 0.05 and log_2_ FC (fold change) > 1. Volcano plots and heatmaps were generated to overview the gene expression profiles by using the R package.

### Pathway and process enrichment analyses of differentially expressed genes

For the DEGs from each comparison, pathway and process enrichment analyses were performed by using the Metascape (http://metascape.org/) (Zhou et al., 2019). The analyses were based on the following databases: GO Biological Processes, KEGG Pathways, Reactome Gene Sets, and Canonical Pathways. Terms with count ≥3, *p*-value < 0.01, and enrichment factor (observed counts/counts expected by chance) >1.5 were grouped in clusters based on the similarities. Specifically, *p*-values were calculated based on the accumulative hypergeometric distribution, and q-values were calculated by using the Banjamini-Hochberg procedure for multiple testing (Hochberg and Benjamini, 1990). Hierarchical clustering was performed on the enriched terms with the kappa scores, and sub-trees with a similarity >0.3 were considered a cluster (Roberts, 2008). The most statistically significant term within a cluster was used to represent the cluster. The overlaps between three gene lists were shown in a Circos plot (Krzywinski et al., 2009). Heatmaps of the top 20 and 100 enriched terms were generated by using the JTreeView (Saldanha, 2004).

### Networks of significantly enriched terms

To further investigate the relationships among the enriched terms, network plots of the top 20 clusters were generated, where terms with a similarity >0.3 were connected by edges. The networks were visualized by using the Cytoscape software v3.7.2 (https://cytoscape.org/), where each node represented an enriched term and was colored by its cluster ID and then by *p*-value (Shannon et al., 2003). In addition, the nodes were represented as pie charts, where the size of a pie was proportional to the total number of hits belonging to the specific term. The pie charts were color-coded based on the gene list identities, and the size of a slice showed the percentage of genes under the term which originated from the specific gene list.

### Protein–protein interaction network of differentially expressed genes and modular analysis

For all DEGs, PPI network analysis was carried out by using the Metascape. The analyses were based on several databases, including BioGrid, InWeb_IM, and OmniPath (Stark et al., 2006; Turei et al., 2016; Li et al., 2017). The resultant network contained the subset of proteins with at least one physical interaction with one other member. The Molecular Complex Detection (MCODE) algorithm was applied to identify highly connected network components (Bader and Hogue, 2003). The PPI network plots were visualized by using the Cytoscape, where each node represented a hub protein and was colored by the cluster ID and gene list identity.

### Functional enrichment of 26 key genes

For the 26 key genes identified in MOCDE 1, GO enrichment analyses of biological process (BP), molecular function (MF), and cellular component (CC) were conducted by using the gProfiler (https://biit.cs.ut.ee/gprofiler/) (Raudvere et al., 2019). In addition, KEGG pathway analysis was performed to analyze significantly enriched signaling pathways. A *p*-value < 0.05 was applied as the threshold value.

### Protein–protein interaction analysis of 26 key genes

By using the GeneMANIA (http://genemania.org/), a flexible and user-friendly web interface, PPI network and functional assays of 26 key genes were generated and visualized (Zuberi et al., 2013). Several types of relationships among proteins included co-expressions, physical interactions, shared protein domains, pathways, website predictions, co-localizations, and genetic interactions. GeneMANIA also reported weights that indicated the predictive value of each selected data set.

### Gene set enrichment analysis of ischemia and reperfusion samples

GSEA is a computational method used to determine whether a predefined set of genes shows significant and concordant differences between the two study groups. GSEA software v3.0 (http://www.gsea-msigdb.org/gsea/) was used to perform KEGG pathway enrichment for two comparisons: end-ischemia vs. cardiac arrest and reperfusion vs. cardiac arrest (Subramanian et al., 2005). A *p*-value < 0.05 was considered as statistically significant. The “c2.cp.kegg.v6.2.symbols.gmt” gene set was downloaded from the Molecular Signatures Database and used as the reference gene set.

### Myocardial I/R injury in mice

The protocol of animal study was approved by the Ethic Committee for Animal Care and Use at Soochow University, Suzhou, Jiangsu, China. The animal experimental procedures followed the Guide for the Care and Use of Laboratory Animals (National Institutes of Health publication No. 85-23, revised in 1996). Adult male C57BL/6J mice (8 weeks old, 20–25 g) were provided by the Experimental Animal Center of Soochow University. Mice were housed in a controlled condition (temperature of 24–26°C, 12-h light/dark cycle, and free access to food and water).

We induced myocardial I/R injury in mice as previously reported (Peng et al., 2021). First, the mice were anesthetized with intraperitoneal sodium pentobarbital (50 mg/kg). Next, the mice were endotracheally intubated and received mechanical ventilation using a rodent respirator (RWD, Shenzhen, China). A left thoracotomy was performed in the left fourth intercostal space. The left anterior descending coronary artery (LAD) was temporarily ligated at ∼2 mm under the lower edge of the left auricle. Observation of tissue blanching in the left ventricular myocardium and elevation in the ST-segment of electrocardiography indicated that the myocardial I/R model was successfully established. The LAD was occluded for 30 min to induce myocardial ischemia, and the ligature was removed for reperfusion of 30 min. For mice in the sham surgery group, the same surgical procedure was performed except that the suture placed around the LAD was not tied. At the end of the reperfusion, the hearts were harvested.

### Area at risk and infarct size of myocardium

Evans blue staining and 2,3,5-triphenyltetrazolium chloride (TTC) staining were used to assess the area at risk and infarct size of myocardium, as previously described (Peng et al., 2020; Song et al., 2020; Yang et al., 2021). Briefly, the LAD was re-ligated following 30 min of reperfusion, and 1 ml of 2% Evans blue dye (Sigma, St. Louis, MO) was perfused into the coronary artery. The heart was then immediately excised and quickly frozen at −80°C. The heart samples below the ligature were sliced into five transverse sections, and the slices were incubated with 1% TTC (Sigma, St. Louis, MO) at 37°C for 15 min in a dark chamber and fixed with 10% formaldehyde overnight. The non-ischemic area (blue), the myocardium area at risk (AAR, red), and infarct area (IA, pale) were measured using the Image Pro Plus software (Media Cybernetics, Silver Spring, MD, United States). The ratios of IA/AAR and AAR/left ventricular area (LV) were calculated.

### Histological examination of myocardium

Histological structure of the left ventricular myocardium samples was detected using the hematoxylin and eosin (HE) staining (Yang et al., 2021). Briefly, myocardial tissues were fixed with paraformaldehyde and embedded with paraffin. The samples were cut transversely into 5 µm-thick sections. After dewaxing with dimethylbenzene and dehydrating with graded ethanol series, the slices were stained with hematoxylin and eosin. Finally, histological damage of the myocardium was observed under a light microscope (Nikon Corporation; Tokyo, Japan).

### Myocardial apoptosis assay

Cell apoptosis in the left ventricular myocardium samples was assessed using the terminal deoxynucleotidyl transferase dUTP nick‐end labeling (TUNEL) analysis with the *in situ* Cell Death Detection kit (Roche Molecular Biochemicals, Mannheim, Germany) (Peng et al., 2020; Peng et al., 2021). The heart samples were fixed in 4% paraformaldehyde, dehydrated, embedded in paraffin, and cut into 4 μm‐thick sections. The slices were incubated with TUNEL reaction mixture and then counterstained with the 4′,6-diamino-2-phenylindole (DAPI) to show the nuclei. At least four random and nonoverlapping of views in areas surrounding the infarct area in the I/R group or the corresponding area in the sham group were observed using a fluorescence microscope (Nikon Corporation; Tokyo, Japan). The apoptosis rate was expressed as a percentage of TUNEL-positive cells to total cell nuclei.

### Quantitative real-time polymerase chain reaction

Total RNA in the left ventricular myocardium samples was extracted using the TRIZOL reagent (Thermo Fisher Scientific, Waltham, MA). After RNA quantification and purity evaluation, reverse transcription was conducted with a cDNA Synthesis Kit (abm, Richmond, BC, Canada), in a 20 μl reaction system containing 5× All-in-one, DEPC water, and RNA samples. Then, qPCR was performed with the EvaGreen (abm, Richmond, BC, Canada) on the Roche Light Cycler R480 System (Roche, Bedford, MA). The amplification conditions were: Pre-denaturation at 93°C for 10 s, denaturation at 55°C for 15 s, and annealing at 72°C for 20 s for 40 cycles. Three replicates were used for each sample. Gene expression were analyzed by using the 2^−ΔΔCT^ method and normalized to β-tubulin. The sequences of primers (Sangon Biotech, Shanghai, China) are listed in Table 1.

**TABLE 1:** Specific primers.

Oligo	Sequence (5′–3′)
EDN1-F	TTT TCC CGT GAT CTT CTC TCT G
EDN1-R	CAG AAG TAG ACA CAC TCC TTG T
CASR-F	ACA GGT TAC TCA ATA GCT CCA C
CASR-R	GTA CAC GTT GTA GGA TAT CCG T
XCR1-F	GGG TCT TGG TGA AGT ATG AGA A
XCR1-R	CTT GCA GAA GAA GTC ACC TAG A
CCL21-F	TTT TTA CCA AGT GGC CTC TGA A
CCL21-R	GCG TTG ATT TCA AAG TGG GTA A
CXCL13-F	TG TGA TCT GGA CCA AGA TGA A
CXCL13-R	GAC TTT TGC TTT GGA CAT GTC T

### Western blots

The total protein of the left ventricular myocardium samples was extracted using the RIPA reagents (Beyotime, Shanghai, China). The protein concentrations were detected using a bicinchoninic acid protein assay kit (Beyotime, Shanghai, China). All proteins were separated with 10% sodium dodecyl sulphate-polyacrylamide gel electrophoresis, and transferred onto polyvinylidene fluoride membranes (Millipore Corp., Bedford, MA). The membranes were incubated with the primary antibodies overnight at 4°C, and then incubated with the horseradish peroxidase-conjugated secondary antibodies for 2 h at room temperature. The primary antibodies were as follows: goat anti-CCL21 (1:1000; R&D Systems, Minneapolis, MN), rabbit anti-XCR1 (1:1000; Novus Biologicals, Littleton, CO), goat anti-CXCL13 (1:1000; R&D Systems, Minneapolis, MN), goat anti-CCR7 (1:1000; Novus Biologicals, Littleton, CO), rabbit anti-GPR174 (1:1000; Biorbyt, Cambridge, United Kingdom), rabbit anti-CXCR5 (1:1000; Absin, Shanghai, China), and rabbit anti-β-tubulin (1:5000; Cell Signaling Technology, Beverly, MA). Finally, the protein bands were detected using the Tanon 5200 luminescent workstation (Tanon, Shanghai, China). The protein expression was normalized to β-tubulin as control.

### Immunofluorescence staining

Following 30 min of reperfusion, the left ventricular myocardium samples were fixed with 10% formalin and embedded in paraffin. Then, the tissues were cut transversely into 5 µm-thick sections, deparaffinized, rehydrated, and subjected to antigen retrieval. For immunofluorescence staining, the slices were incubated with primary antibodies overnight at 4°C, and then incubated with secondary antibodies for 2 h at room temperature. The following primary antibodies were used: goat anti-CCR7 (1:200; Novus Biologicals, Littleton, CO), rabbit anti-GPR174 (1:200; Biorbyt, Cambridge, United Kingdom), mouse anti-α-Actinin (1:250; Santa Cruz Technology, CA), and mouse anti-Vimentin (1:200; Abcam, CA). The nuclei were counterstained with DAPI. At least four random and nonoverlapping of views in areas surrounding the infarct area in the I/R group or the corresponding area in the sham group were observed using the fluorescence microscope. Images were analyzed using the Image Pro Plus software.

### Statistical analysis

Statistical analysis was performed by using the GraphPad Prism software (version 9.0, GraphPad, San Diego, CA). Data were expressed as mean ± standard error of the mean and compared using *t*-test. A two-tailed value of *p* < 0.05 was considered statistically significant.

## Results

### Identification of differentially expressed genes during myocardial I/R injury

**FIGURE 1:**
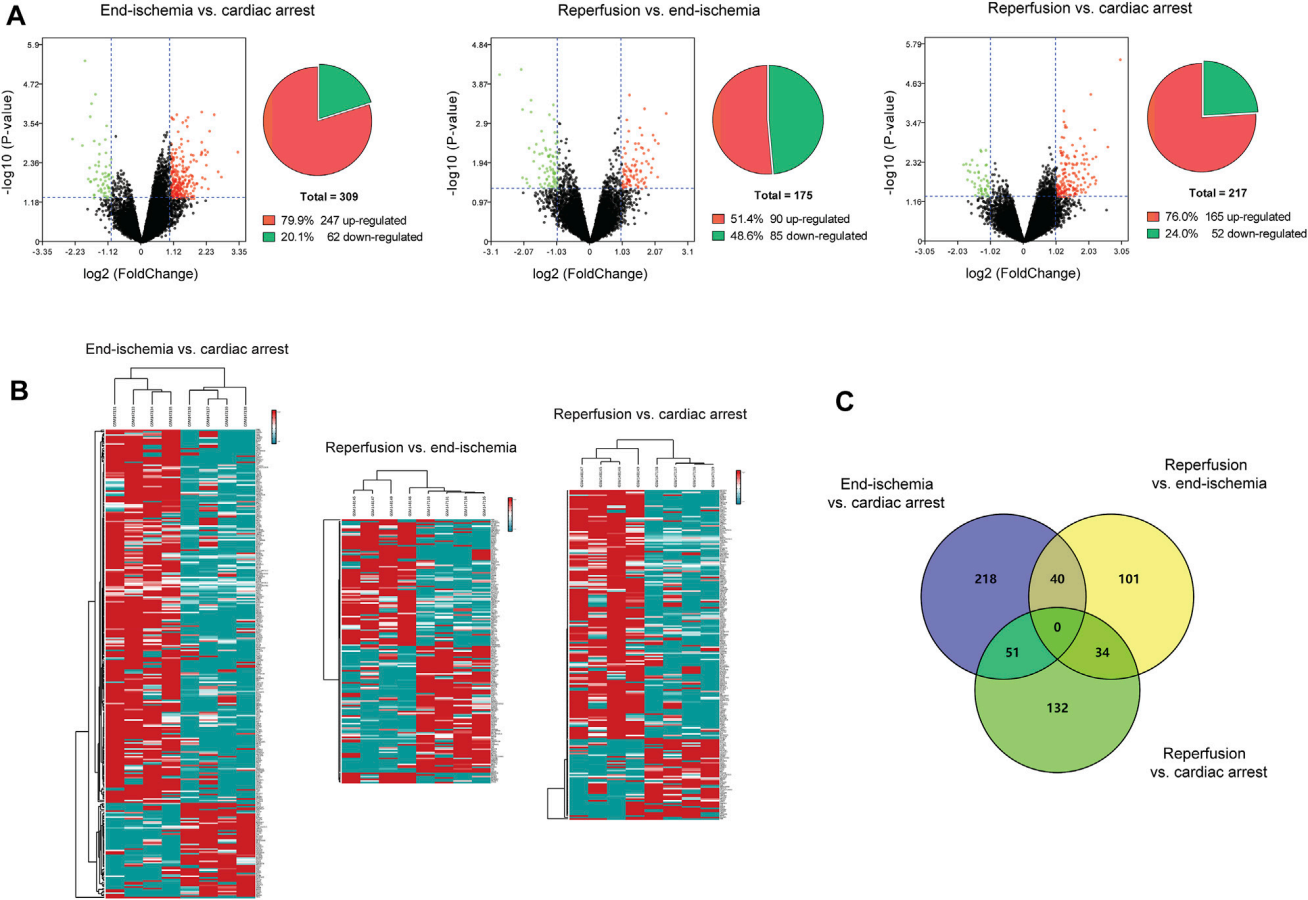
Identification of DEGs. **(A)** Volcano plots and pie charts showing DEGs for end-ischemia vs. cardiac arrest, reperfusion vs. end-ischemia, and reperfusion vs. cardiac arrest. **(B)** Heatmaps of DEGs. **(C)** Venn’s diagram showing common DEGs among comparisons.

The expression profiles of 12,549 genes in GSE6381 of human right ventricular samples during myocardial I/R process were analyzed. The DEGs were identified in three comparisons (Figure 1A): 309 DEGs (247 upregulated and 62 downregulated) in end-ischemia vs. cardiac arrest (Supplementary Table S1), 175 DEGs (90 upregulated and 85 downregulated) in reperfusion vs. end-ischemia (Supplementary Table S2), and 217 DEGs (165 up-regulated and 52 down-regulated) in reperfusion vs. cardiac arrest (Supplementary Table S3). These DEGs showed apparently differential expression profiles (Figure 1B). The top 10 up- and downregulated DEGs for each comparison are presented in Table 2. There were 40, 34, and 51 common DEGs between two comparisons, but no gene was found as common to the three (Figure 1C; Supplementary Table S4).

**TABLE 2:** Top 10 upregulated and downregulated DEGs for each comparison.

	Upregulated	log2FC	p-value	Downregulated	log2FC	p-value
End-ischemia vs. cardiac arrest	CUX2	3.336828	1.25E-06	C3orf36	−2.3478	0.000816
	TPH1	3.265775	0.001979	SUSD5	−2.02744	0.001272
	KPNA5	2.703	0.010906	SLC15A3	−1.93057	3.68E-06
	KIF23	2.593434	0.007537	FBXL15	−1.8243	0.012578
	FCGR1A	2.45906	0.000147	ST6GAL1	−1.76525	0.005889
	KRT5	2.301802	0.000472	EPHX2	−1.75484	0.014952
	GABRA6	2.264973	0.024899	ENTPD7	−1.74397	0.000172
	TEX12	2.246953	0.001967	PARP11	−1.72645	0.01515
	MDM4	2.198717	0.004825	UBIAD1	−1.7032	6.85E-05
	NPY1R	2.175747	0.002343	VASH2	−1.62708	0.037364
Reperfusion vs. end-ischemia	THAP9	2.392984	0.000677	SPICE1	−2.86651	7.54E-05
	KLF3-AS1	2.31404	1.45E-05	MDM4	−2.17014	5.58E-05
	BARD1	2.143729	0.003722	RAG1	−2.14513	0.000531
	NEUROD1	2.117173	0.025112	SPANXB1	−2.13357	0.025505
	ZNF124	2.0379	0.003054	SPATA1	−2.00897	0.027544
	SLC5A12	1.968573	0.008834	GABRA6	−1.98757	0.035172
	BMPR1B	1.967616	0.002339	SRY	−1.9426	0.004901
	CDIP1	1.92239	0.01451	TRHDE	−1.91529	0.04149
	MATN3	1.919535	0.018881	MMP17	−1.86525	0.000624
	MNX1	1.832299	0.012009	LOC389906	−1.8647	0.000316
Reperfusion vs. cardiac arrest	KPNA5	3.054913	0.010476	ZNF667	−1.87117	0.005366
	CUX2	2.981681	4.32E-06	NPL	−1.81029	1.62E-06
	THAP9	2.600435	0.001636	SSSCA1	−1.79372	0.005242
	JPH3	2.232684	0.003121	GSR	−1.7579	0.014834
	CSN1S1	2.225056	0.015934	GTF3C5	−1.73627	0.008104
	SLC5A12	2.203091	0.01966	CTB-176F20.3	−1.67732	0.008956
	TRPM6	2.184206	0.000506	LTBR	−1.66943	0.005418
	MBL2	2.170036	0.01262	RAG1	−1.62025	0.003965
	CCDC81	2.128599	0.003659	BTF3P11	−1.61682	0.017243
	MTNR1A	2.10746	0.013072	EMILIN2	−1.54244	0.009152

DEG, differentially expressed gene; FC, fold change.

### Pathway and process enrichment analyses of differentially expressed genes revealed significantly enriched terms

**FIGURE 2:**
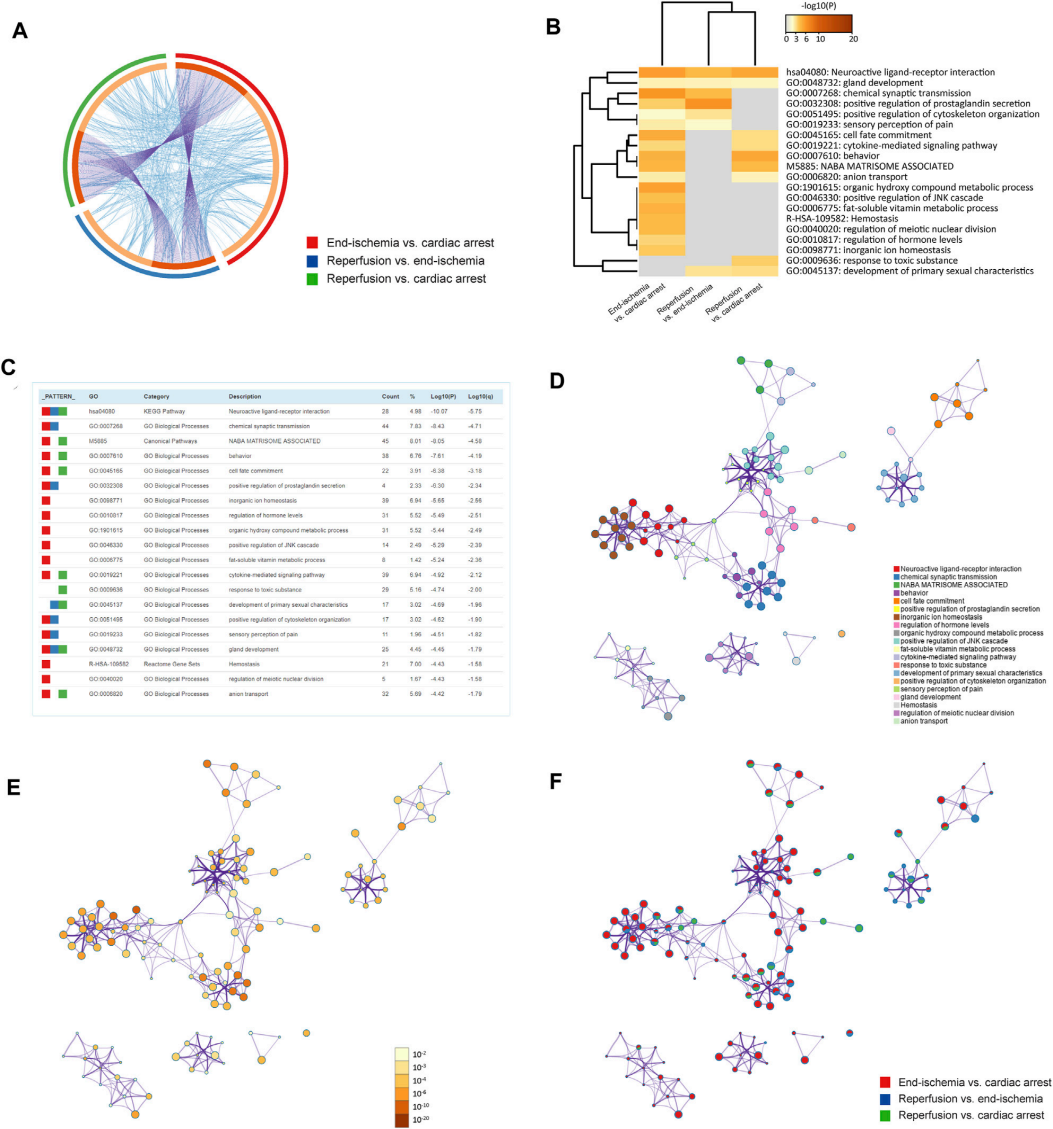
Functional enrichment analyses of DEGs. **(A)** Circos plot showing gene overlaps and genes of the same enriched ontology term. Purple curves link common genes, and blue curves link genes belonging to the same enriched term. Dark orange indicates genes that hit multiple lists, and light orange indicates genes unique to a list. **(B)** Heatmap showing top 20 enriched terms, colored by *p*-values. **(C)** Characteristics of top 20 enriched terms. Count (%) indicates the number (percentage) of genes in the given ontology term. **(D)** Networks colored by cluster IDs, where nodes with the same ID are close to each other. **(E)** Networks colored by *p*-values. **(F)** Networks with pie charts, color-coded by identities of the genes.

The pathway and process enrichment analyses of DEGs for each comparison were conducted by using the Metascape. Figure 2A shows gene overlaps and genes that belong to the same enriched ontology term. The top 20 enriched terms are colored by *p*-values in the heatmap (Figure 2B), with the characteristics shown in Figure 2C. Of these, inorganic ion homeostasis, positive regulation of JNK cascade, and cytokine-mediated signaling pathway may be closely related to the myocardial I/R process. Neuroactive ligand-receptor interaction is the common KEGG pathway. The top 100 enriched terms are available in Supplementary Figure S1. Furthermore, the relationships of the significantly enriched terms are visualized in networks, colored by cluster IDs (Figure 2D), *p*-values (Figure 2E), and gene list identities (Figure 2F). All results of the enrichment analyses are listed in Supplementary Table S5.

### Protein–protein interaction analyses of differentially expressed genes revealed key modules and hub proteins

**FIGURE 3:**
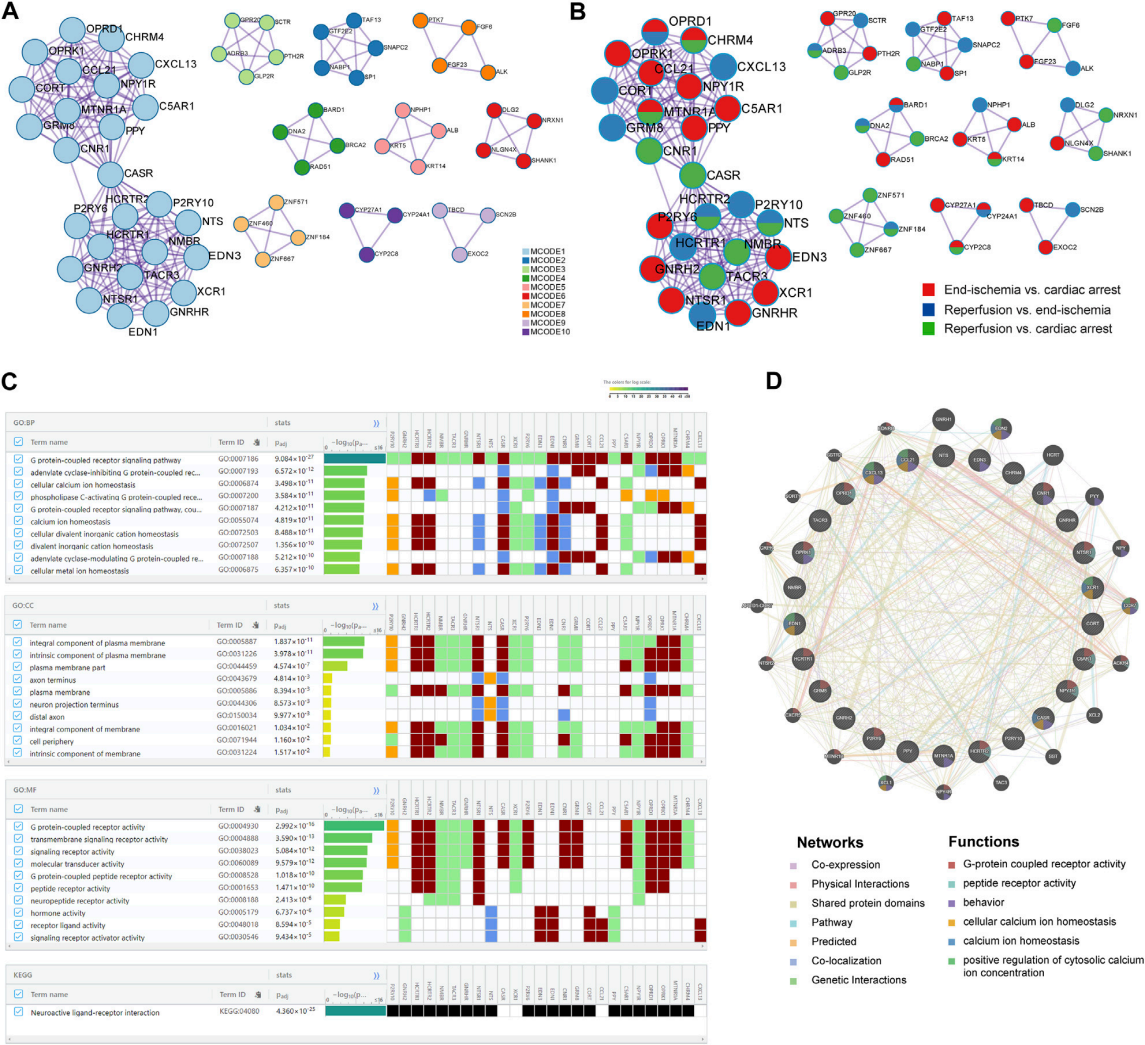
Key candidate genes and PPI network analysis. **(A)** Interactions among hub proteins in MCODE cluster 1. **(B)** Interactions among hub proteins, colored by identities of the proteins. **(C)** Top 10 enriched BP, CC, and MF terms, and enriched KEGG pathway. **(D)** Protein-protein interaction networks and functions.

The PPI networks of DEGs were performed to identify key modules with highly clustered proteins using the MCODE functionality. All interactions of the proteins are presented in Supplementary Figure S2. The PPI analyses revealed a total of 10 key modules. The MCODE one contained 26 highly clustered proteins: C5AR1, CASR, CCL21, CHRM4, CNR1, CORT, CXCL13, EDN1, EDN3, GNRH2, GNRHR, GRM8, HCRTR1, HCRTR2, MTNR1A, NMBR, NPY1R, NTS, NTSR1, OPRD1, OPRK1, P2RY10, P2RY6, PPY, TACR3, and XCR1. In the MCODE networks, the interactions among the 26 hub proteins were colored by MCODE cluster IDs (Figure 3A) and identities of the proteins (Figure 3B). The annotations of the 26 genes are presented in Table 3.

**TABLE 3:** Annotation of 26 key genes.

Gene	Gene ID	Species	Description	Gene location
*C5AR1*	728	*H. sapiens*	complement C5a receptor 1	chr19:47309861-47322065
*CASR*	846	*H. sapiens*	calcium sensing receptor	chr3:122183668-122291628
*CCL21*	6366	*H. sapiens*	C-C motif chemokine ligand 21	chr9:34709005-34710136
*CHRM4*	1132	*H. sapiens*	cholinergic receptor muscarinic 4	chr11:46383791-46391614
*CNR1*	1268	*H. sapiens*	cannabinoid receptor 1	chr6:88139866-88163023
*CORT*	1325	*H. sapiens*	cortistatin	chr1:10450031-10451998
*CXCL13*	10,563	*H. sapiens*	C-X-C motif chemokine ligand 13	chr4:77511753-77611836
*EDN1*	1906	*H. sapiens*	endothelin 1	chr6:12290361-12297194
*EDN3*	1908	*H. sapiens*	endothelin 3	chr20:59300611-59325992
*GNRH2*	2797	*H. sapiens*	gonadotropin releasing hormone 2	chr20:3043622-3045747
*GNRHR*	2798	*H. sapiens*	gonadotropin releasing hormone receptor	chr4:67737381-67756086
*GRM8*	2918	*H. sapiens*	glutamate metabotropic receptor 8	chr7:126438598-127243515
*HCRTR1*	3061	*H. sapiens*	hypocretin receptor 1	chr1:31617700-31627318
*HCRTR2*	3062	*H. sapiens*	hypocretin receptor 2	chr6:55106460-55282741
*MTNR1A*	4543	*H. sapiens*	melatonin receptor 1A	chr4:186533655-186555567
*NMBR*	4829	*H. sapiens*	neuromedin B receptor	chr6:142074484-142088799
*NPY1R*	4886	*H. sapiens*	neuropeptide Y receptor Y1	chr4:163323962-163332596
*NTS*	4922	*H. sapiens*	neurotensin	chr12:85874295-85882992
*NTSR1*	4923	*H. sapiens*	neurotensin receptor 1	chr20:62708836-62762771
*OPRD1*	4985	*H. sapiens*	opioid receptor delta 1	chr1:28812170-28871267
*OPRK1*	4986	*H. sapiens*	opioid receptor kappa 1	chr8:53225724-53251637
*P2RY10*	27334	*H. sapiens*	P2Y receptor family member 10	chrX:78945391-78963727
*P2RY6*	5031	*H. sapiens*	pyrimidinergic receptor P2Y6	chr11:73264505-73298625
*PPY*	5539	*H. sapiens*	pancreatic polypeptide	chr17:43940804-43942476
*TACR3*	6870	*H. sapiens*	tachykinin receptor 3	chr4:103586033-103719985
*XCR1*	2829	*H. sapiens*	X-C motif chemokine receptor 1	chr3:46017009-46027483

### Identification of key candidate genes related to calcium homeostasis and regulation

Based on the 26 genes, GO and KEGG enrichment analyses were performed using the gProfiler. Of the top significantly enriched BP, CC, and MF terms, cellular calcium ion homeostasis (GO:0006874) and calcium ion homeostasis (GO:0055074) were noted in the process of myocardial I/R injury (Figure 3C). Thirteen genes were involved, including *P2RY10*, *HCRTR1*, *HCRTR2*, *NTSR1*, *CASR*, *XCR1*, *P2RY6*, *EDN3*, *EDN1*, *CNR1*, *CCL21*, *C5AR1*, and *CXCL13*. In addition, KEGG analysis showed one enriched pathway, neuroactive ligand-receptor interaction (Figure 3C), in line with the previous results (Figure 2C). All enrichment results are available in Supplementary Figure S3; Supplementary Table S6.

To further reveal the interactions of the 26 hub proteins, PPI network and functional analysis were conducted using the GeneMANIA (Figure 3D). There were substantial and complex relationships within these proteins, including co-expressions, physical interactions, shared protein domains, pathways, predicted interactions, co-localizations, and genetic interactions (Supplementary Table S7). In addition, the functional analysis revealed several gene functions (Supplementary Table S8). Of note, there were three functions of calcium regulation (cellular calcium ion homeostasis, calcium ion homeostasis, and positive regulation of cytosolic calcium ion concentration). In these calcium-related functions, five hub proteins (CCL21, XCR1, CXCL13, EDN1, and CASR) were revealed, which were significantly enriched in two calcium processes (Figure 3C).

### Gene set enrichment analysis of ischemia and reperfusion samples revealed significantly enriched pathways

**FIGURE 4:**
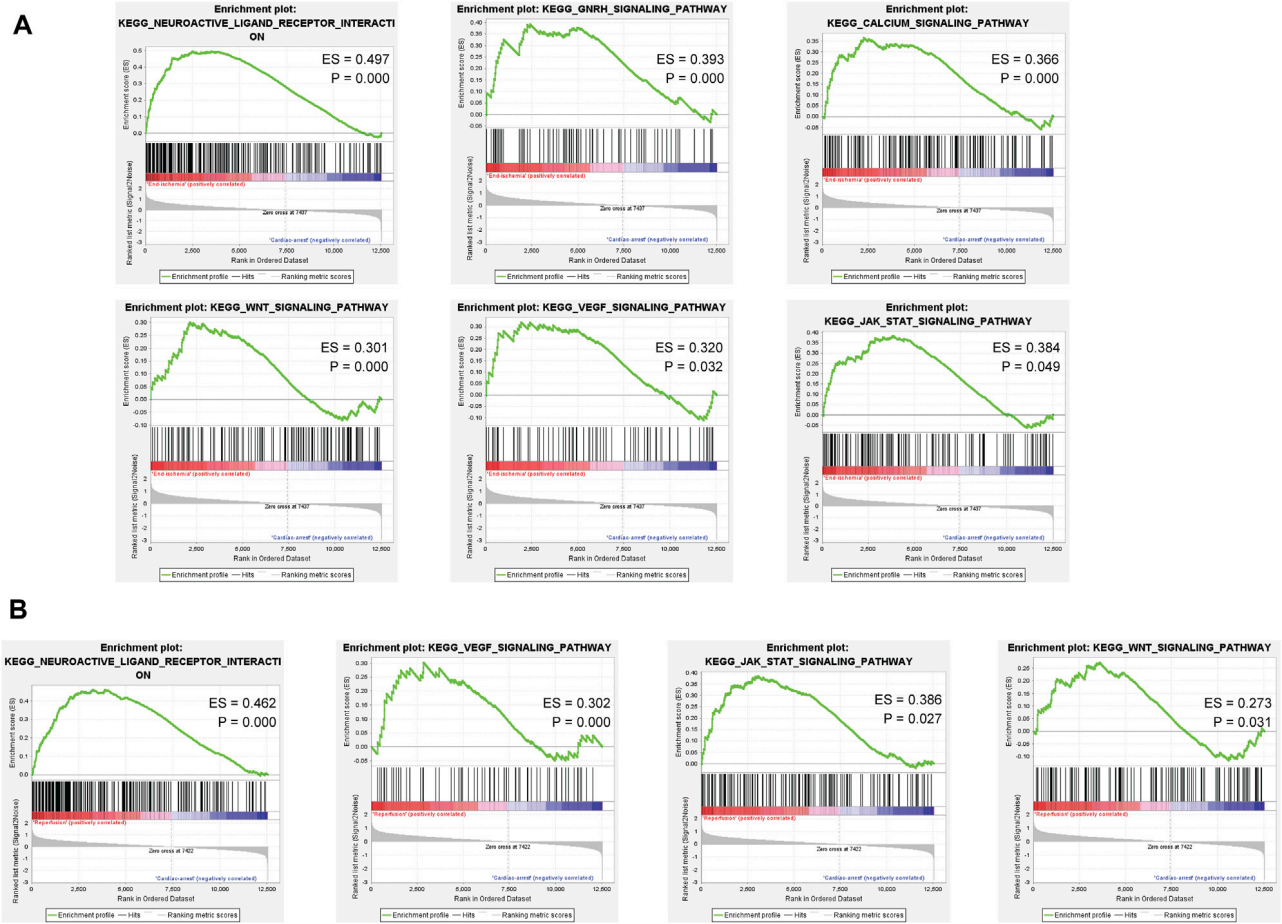
Gene set enrichment analysis of the ischemia and reperfusion samples. **(A)** Enriched pathways in ischemia samples. **(B)** Enriched pathways in reperfusion samples.

Based on the ischemia samples, GSEA revealed several significantly enriched pathways, including neuroactive ligand receptor interaction, GNRH signaling pathway, calcium signaling pathway, WNT signaling pathway, VEGF signaling pathway, and JAK-STAT signaling pathway (Figure 4A). Based on the reperfusion samples, GSEA revealed several significantly enriched pathways, including neuroactive ligand receptor interaction, VEGF signaling pathway, JAK-STAT signaling pathway, and WNT signaling pathway (Figure 4B). All results of GSEA enriched pathways are available in Supplementary Tables S9, S10.

### Pathological changes and apoptosis in myocardial I/R injury

**FIGURE 5:**
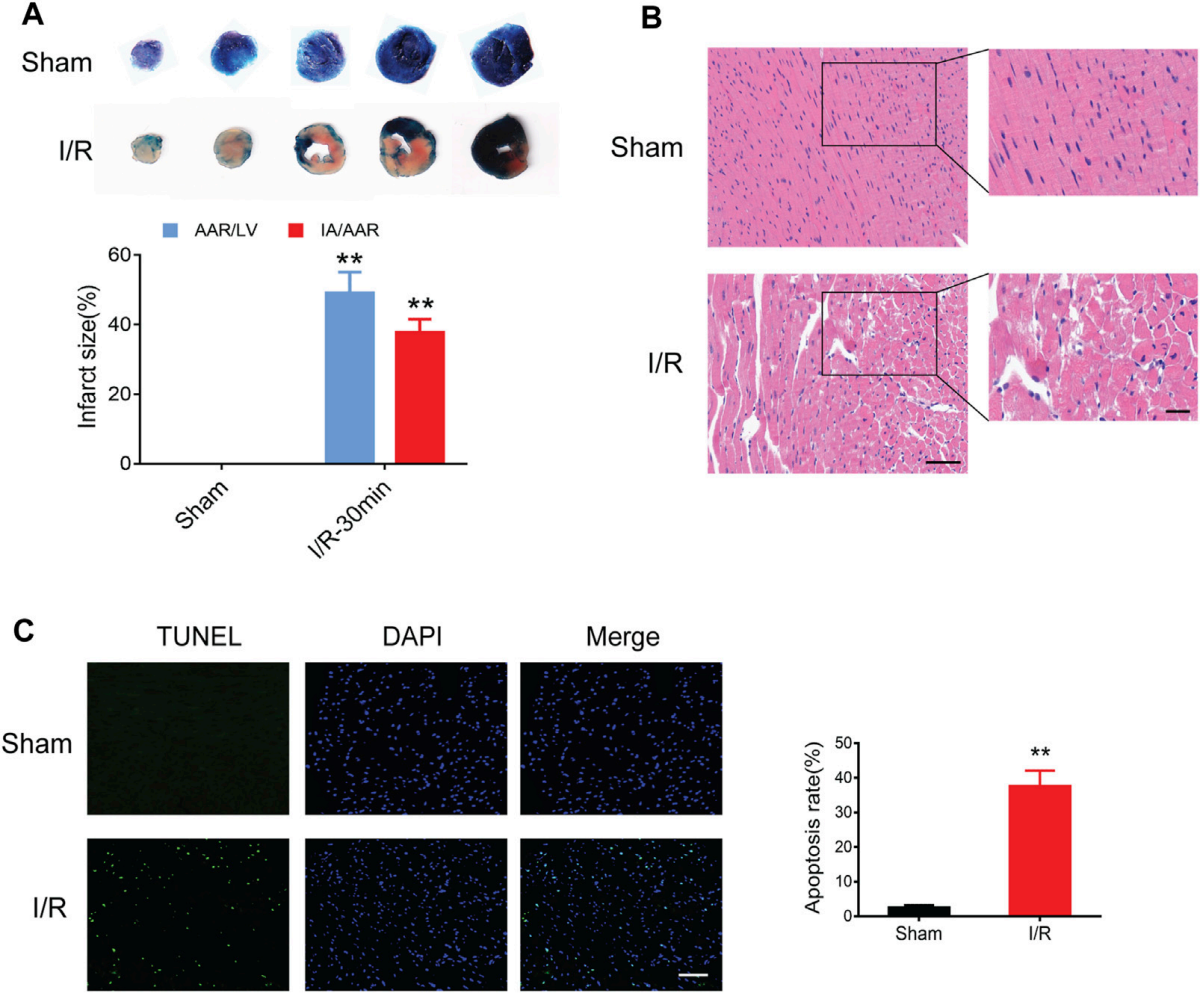
Myocardial I/R injury in mice. **(A)** Evans blue/TTC staining showing myocardial infract size in the sham and I/R groups. **(B)** HE staining showing myocardial histological damage. Scale bar = 100 μm (left) and 50 μm (right). **(C)** TUNEL staining showing the apoptosis rate in the myocardium. Scale bar = 100 μm *n* = 3. **p < 0.01 vs. the sham group. I/R, ischemia reperfusion; AAR, area at risk; IA, infarct area; LV, left ventricle.

Compared to the sham controls, myocardial I/R injury led to an obvious myocardial infract size (approximately 40%) in the Evans blue/TTC staining (Figure 5A). In addition, HE staining showed remarkable myocardial histological damage following myocardial I/R injury (Figure 5B). The structure of myocardial tissue in the sham group was clear and aligned, with intact myocardial fibers; however, the I/R group showed a damaged tissue structure, swollen cardiomyocytes, disarranged myofibrils, and leukocytes infiltration. The TUNEL assay was performed to assess the level of DNA fragmentation in myocardial apoptotic cells. The results showed a significantly increased TUNEL apoptosis rate induced by myocardial I/R injury (Figure 5C).

### Validation of mRNA and protein expression of key genes and related receptors

**FIGURE 6:**
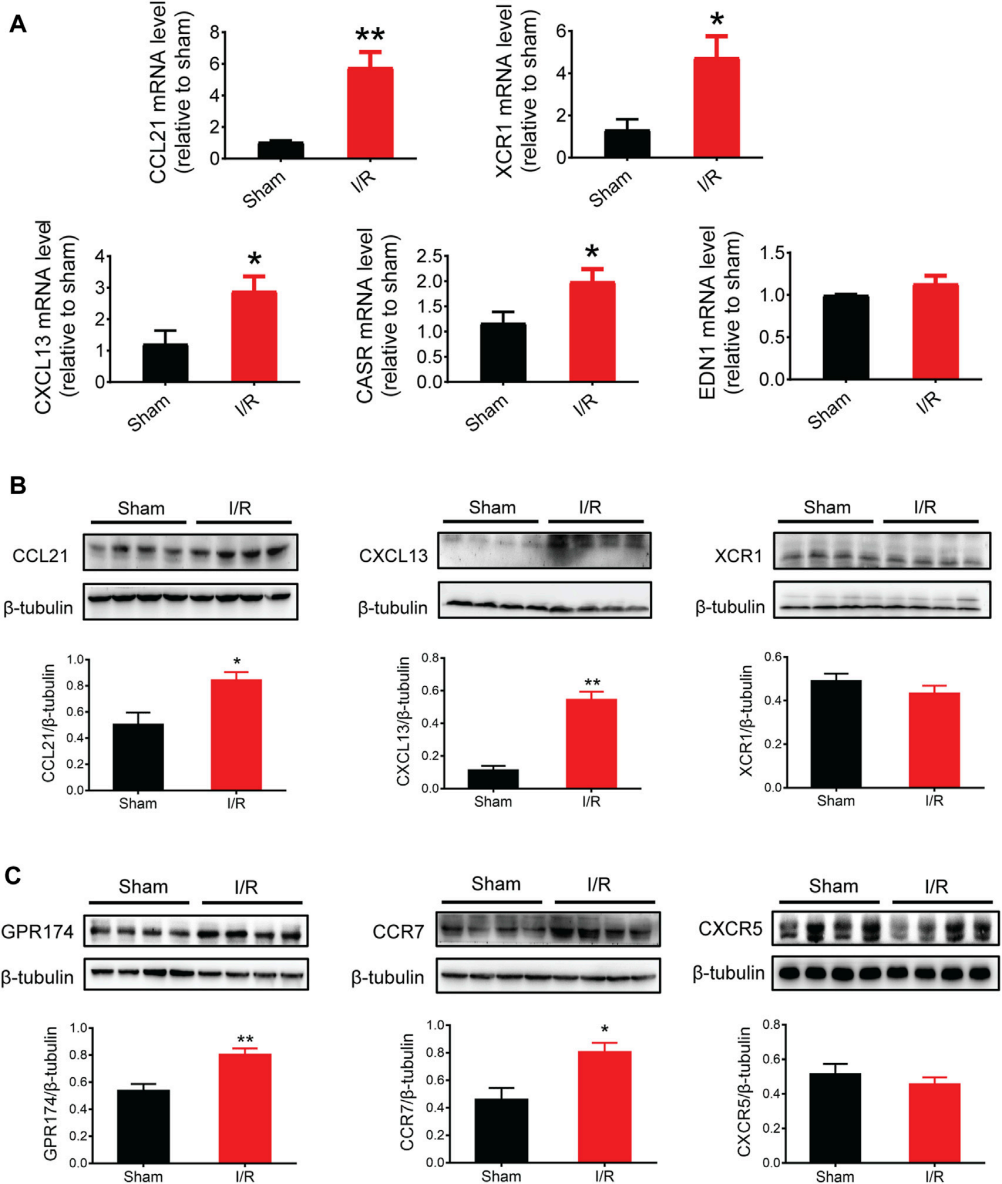
mRNA and protein expression of key genes and related receptors. **(A)** mRNA expression of CCL21, XCR1, CXCL13, CASR, and EDN1. **(B)** Protein expression of CCL21, CXCL13, and XCR1. **(C)** Protein expression of GPR174/CCR7 (the receptors of CCL21) and CXCR5 (the receptors of CXCL13). *n* = 3–6. *p < 0.05, **p < 0.01 vs. the sham group. I/R, ischemia reperfusion.

Based on the PPI network and hub protein analyses, five key genes (*CCL21*, *XCR1*, *CXCL13*, *EDN1*, and *CASR*) were identified. To investigate the role of these genes at the early stage of myocardial I/R injury, the mRNA expression of these genes was determined using the qPCR. The results showed that the mRNA expression of *CCL21*, *XCR1*, *CXCL13*, and *CASR* were significantly up-regulated in the left ventricular samples of myocardial I/R injury (Figure 6A).

Given that chemokines are small secreted proteins with chemoattractant properties that play a key role in inflammation and cell apoptosis (Jarrah et al., 2018; Zhang et al., 2020), we assessed the protein expression of CCL21, XCR1, and CXCL13. The results showed that the protein expression of CCL21 and CXCL13 was significantly increased following myocardial I/R injury (Figure 6B). Next, we tested the protein expression of GPR174 and CCR7 (known receptors of CCL21) (Chen et al., 2020; Van Raemdonck et al., 2020; Zhao et al., 2020) and CXCR5 (a known receptor of CXCL13) (Hussain et al., 2019; Shen et al., 2020). We found that the protein expression of GPR174 and CCR7, but not CXCR5, was significantly elevated. These results suggested that the CCL21-GPR174/CCR7 chemokine axis was involved in the early stage of myocardial I/R injury (Figure 6C).

### Activation of GPR174/CCR7 on cardiac fibroblasts in myocardial I/R injury

**FIGURE 7:**
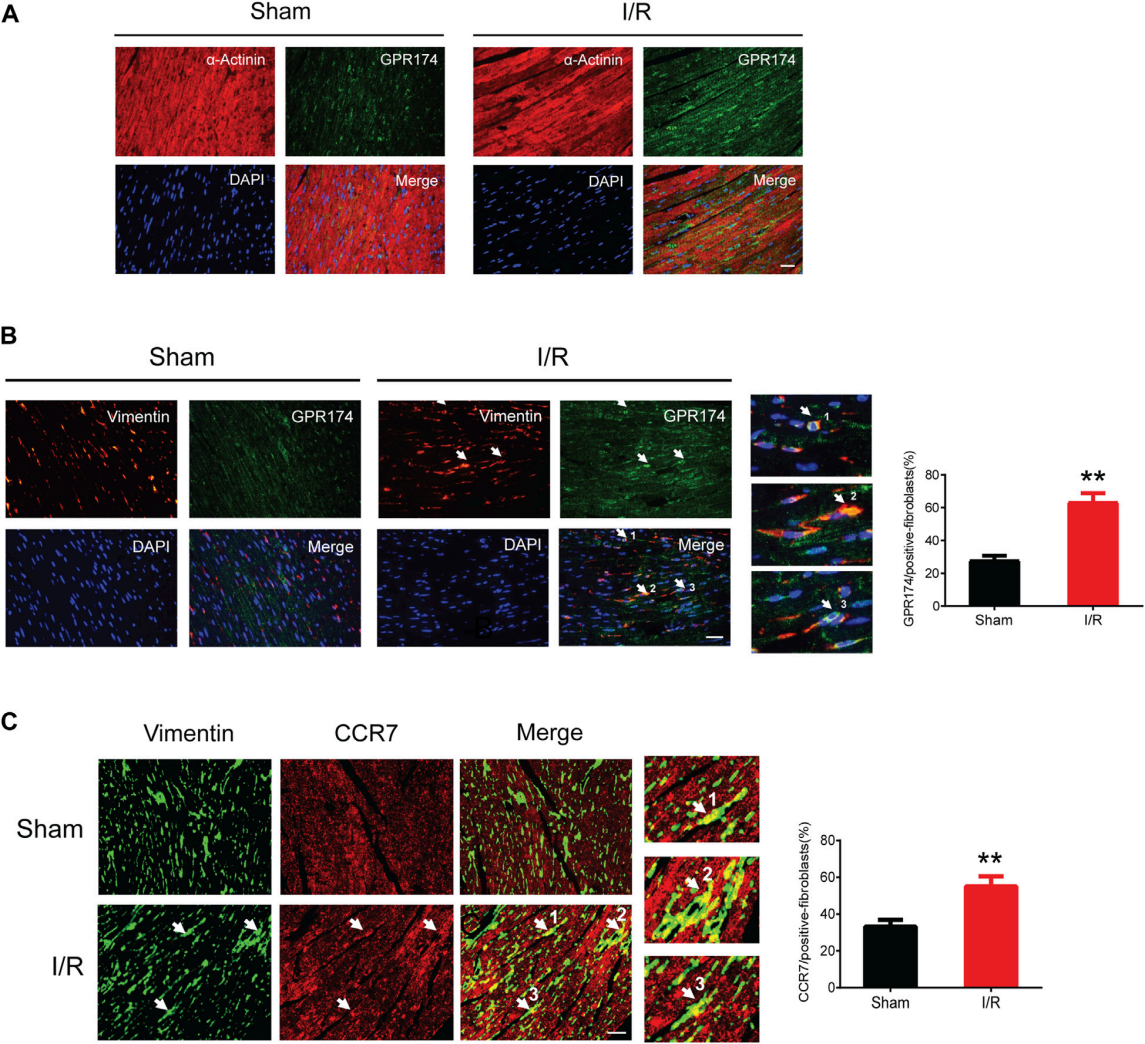
Expression of GPR174 and CCR7 on cardiac fibroblasts during myocardial I/R injury. **(A)** Immunofluorescence staining showing that GPR174 did not present on cardiomyocytes in the sham or I/R groups. **(B)** Immunofluorescence staining showing the expression of GPR174 on cardiac fibroblasts. **(C)** Immunofluorescence staining showing the expression of CCR7 on cardiac fibroblasts in the sham and I/R groups. Scale bar = 50 μm *n* = 3. **p < 0.01 vs. the sham group. I/R, ischemia reperfusion.

We detected the expression and cellular localization of GPR174 and CCR7 using the immunofluorescence staining. The double-labeling immunofluorescence results showed that there was no GPR174 positive cells (green) co-localized with the α-Actinin positive cells (red) in the sham or myocardial I/R mice, suggesting that GPR174 did not present on cardiomyocytes (Figure 7A). Therefore, we further investigated whether GPR174 was expressed in the cardiac fibroblasts. Following myocardial I/R injury, GPR174 positive cells (green) and Vimentin positive cells (red) co-localized in the left ventricular myocardial tissues, and the expression of GPR174 on cardiac fibroblasts was significantly increased (Figure 7B). Similarly, we also found that myocardial I/R-induced activation of CCR7 was also evident on cardiac fibroblasts (Figure 7C). Taken together, these results suggested that the up-regulated GPR174 and CCR7 on the cardiac fibroblasts underlie the pathological course of myocardial I/R injury.

## Discussion

In this study, the gene expression profiles of human right ventricular samples during myocardial I/R process in cardiac surgery was explored with the use of a comprehensive bioinformatics approach. We identified 309 DEGs for end-ischemia vs. cardiac arrest, 175 DEGs for reperfusion vs. end-ischemia, and 217 DEGs for reperfusion vs. cardiac arrest. Based on pathway and process enrichment analyses, PPI analysis, and MCODE assay, several key modules with hub proteins were revealed. Further enrichment and network analyses of the highly clustered proteins highlighted five key candidate genes (*CCL21*, *XCR1*, *CXCL13*, *EDN1*, and *CASR*) related to calcium homeostasis and regulation. In addition, GSEA confirmed several significantly enriched pathways in the ischemia and reperfusion stages. Next, we performed the validation experiments in animal studies using qPCR and Western blots. The results confirmed that the mRNA and protein expression of CCL21 and CXCL13 were elevated during myocardial I/R injury in mice. Furthermore, myocardial I/R injury induced the activation of GPR174/CCR7 (the CCL21 receptors), but not CXCR5 (the CXCL13 receptor). Notably, the increased expression of GPR174/CCR7 was located within cardiac fibroblasts.

The major difference between our study and the previous one (Arab et al., 2007) is the discovery of novel key candidate genes associated with calcium signaling pathway during the myocardial I/R process in cardiac surgery. Of note, these key genes (*CCL21*, *XCR1*, *CXCL13*, *EDN1*, and *CASR*) were significantly enriched in the processes of calcium homeostasis and regulation, including cellular calcium ion homeostasis, calcium ion homeostasis, and positive regulation of cytosolic calcium ion concentration. Given that intracellular calcium overload is a critical mechanism for cardiomyocyte apoptosis and cell death during myocardial I/R injury (Garcia-Dorado et al., 2012; Mattiazzi et al., 2015), the identification of these novel genes may provide a new preventive and therapeutic basis for intraoperative cardioprotection.

Of these five genes, *CCL21*, *XCR1*, *CXCL13*, and *EDN1* have yet to be evaluated in the context of myocardial I/R injury. CCL21, chemokine (C-C motif) ligand 21, is a small cytokine belonging to the CC chemokine family. A previous study showed that CCL21 was responsible for ischemic brain damage, while CD93 mediated neuroprotection *via* suppressing neuroinflammation through downregulation of CCL21 (Harhausen et al., 2010). XCR1, X-C motif chemokine receptor 1, is a chemokine receptor belonging to the G protein-coupled receptor superfamily, which induces calcium mobilization (Yoshida et al., 1998). CXCL13, chemokine (C-X-C motif) ligand 13, has recently been found to play an important role in spinal cord I/R injury (Chen et al., 2019). Specifically, knockdown of CXCL13 by small interfering RNA protected the rats against spinal cord I/R injury *via* suppressing the activity of relevant signaling pathways and proinflammatory cytokines (Chen et al., 2019). EDN1, endothelin-1, is a potent vasoconstrictor produced by vascular endothelial cells. In a porcine liver transplant mode, improved viability of damaged liver grafts by oxygenated hypothermic machine perfusion was associated with a reduced EDN1 expression (Vekemans et al., 2011). CASR, a calcium-sensing receptor, has been investigated in myocardial I/R injury in recent studies, suggesting that inhibition of CASR attenuated myocardial I/R injury through suppressing endoplasmic reticulum stress-associated apoptotic signaling pathways (Sun et al., 2017; Liu et al., 2019; Yin et al., 2019).

According to the GSEA results, several calcium-related KEGG pathways were significantly enriched in the human right ventricular samples during the ischemia and reperfusion stages, including calcium signaling pathway, GNRH signaling pathway, WNT signaling pathway, VEGF signaling pathway, and JAK-STAT signaling pathway. These findings suggest their important contribution to the cellular calcium homeostasis and regulation in the myocardial I/R process. It has been highlighted that disorder of calcium homeostasis is a pivotal mechanism underlying myocardial I/R injury (Garcia-Dorado et al., 2012; Mattiazzi et al., 2015; Kristiansen et al., 2017). Our recent study also showed that H9c2 cardiomyocytes exposed to oxygen-glucose deprivation/reoxygenation had decreased cell viability, elevated intracellular calcium levels, changes in calcium-related signaling activity, and increased cell apoptosis, which was alleviated by pretreatment with dexmedetomidine, a selective α2-adrenoceptor agonist offering protective effects against I/R injury (Yuan et al., 2019).

Until recently, the relation between CCR7 and cardiac fibroblasts in context of myocardial injury has not been well elucidated. A previous study suggested the redistribution of chemokine receptor CCR7 from peripheral blood to the infarcted heart during myocardial I/R following primary percutaneous coronary intervention procedures (Hoffmann et al., 2012). Another study suggested that CCL21/CCR7 interactions was involved in left ventricular remodeling during pressure overload in patients with aortic stenosis (Finsen et al., 2014). Based on the existing literature, we assume that CCR7 might migrate from peripheral blood to cardiac fibroblasts with an interaction of CCL21, which plays a role in the etiology of myocardial I/R injury.

This study has several limitations. First, the bioinformatic analyses were based on a relatively small number of human ventricular samples from a single microarray data. An integrated analysis of several datasets might have provided more information and insight. Second, the GEO dataset (GSE6381) and ensuring animal studies were based on different species and different parts of heart: right ventricular of human from the GEO dataset, and left ventricular of mice from the animal studies. Although we performed the qPCR, western blots, and immunofluorescence staining experiments to validate the expression of the key genes, the myocardial I/R injury model in mice was different from the human heart samples during cardiac surgery. Hence, the differences in methodology and species should be taken into consideration when interpreting these data, and more studies are still needed to collaborate our findings. Third, the expression profiles of myocardium during intraoperative myocardial I/R obtained in this study was only on the gene level, without assessment and relationships of non-coding RNAs, such as micro RNAs, circular RNAs, or long non-coding RNAs. Last, we showed that myocardial I/R injury increased the expression of CCL21, along with the activation of GPR174/CCR7 within cardiac fibroblasts, but further functional experiments are still needed for mechanistic explorations.

In conclusion, the present study revealed five key genes (*CCL21*, *XCR1*, *CXCL13*, *EDN1*, and *CASR*) and calcium homeostasis and regulation pathways during myocardial I/R injury. The expression of CCL21 and CXCL13 were validated in myocardial I/R injury of mice. We highlighted the increased expression of CCL21 with the activation of receptors GPR174/CCR7 on cardiac fibroblasts underlying myocardial I/R injury. Our findings may provide insights into development of preventive and therapeutic targets for intraoperative cardioprotection.

## Data Availability

The data presented in the study are deposited in the Jianguoyun/Nutstore repository, available at: https://www.jianguoyun.com/p/Dd2OkgkQxvzPChiIpcMEIAA.
