# Supplementary figures and images for: Activation of CCL21-GPR174/CCR7 on cardiac fibroblasts underlies myocardial ischemia/reperfusion injury
Source: Front Genet. 2022 Sep 9;13:946524. doi: 10.3389/fgene.2022.946524 (PMC9505909; doi:10.3389/fgene.2022.946524)

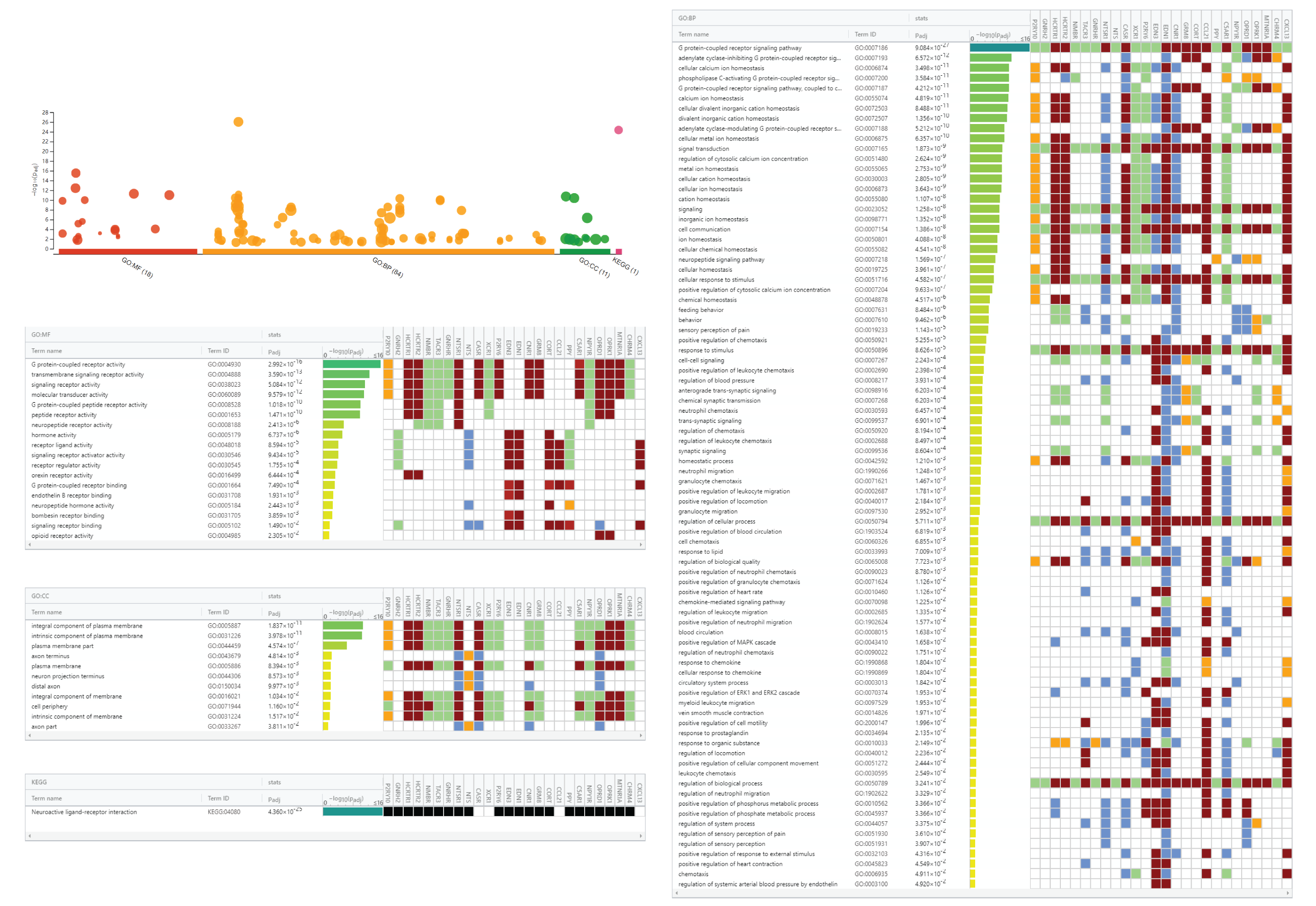

Supplement: Supplementary file 4 [file Image3.TIF]

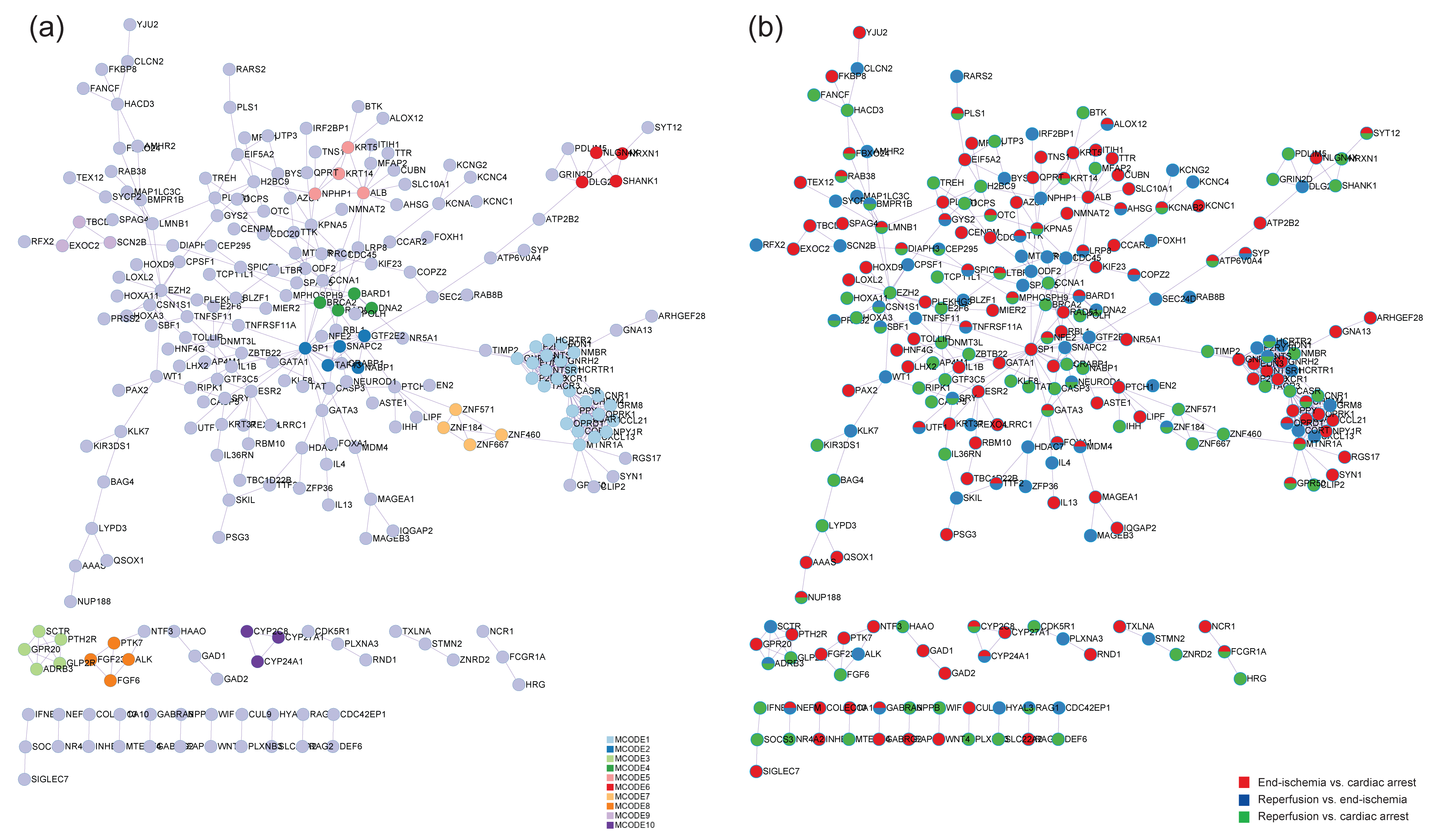

Supplement: Supplementary file 5 [file Image2.TIF]

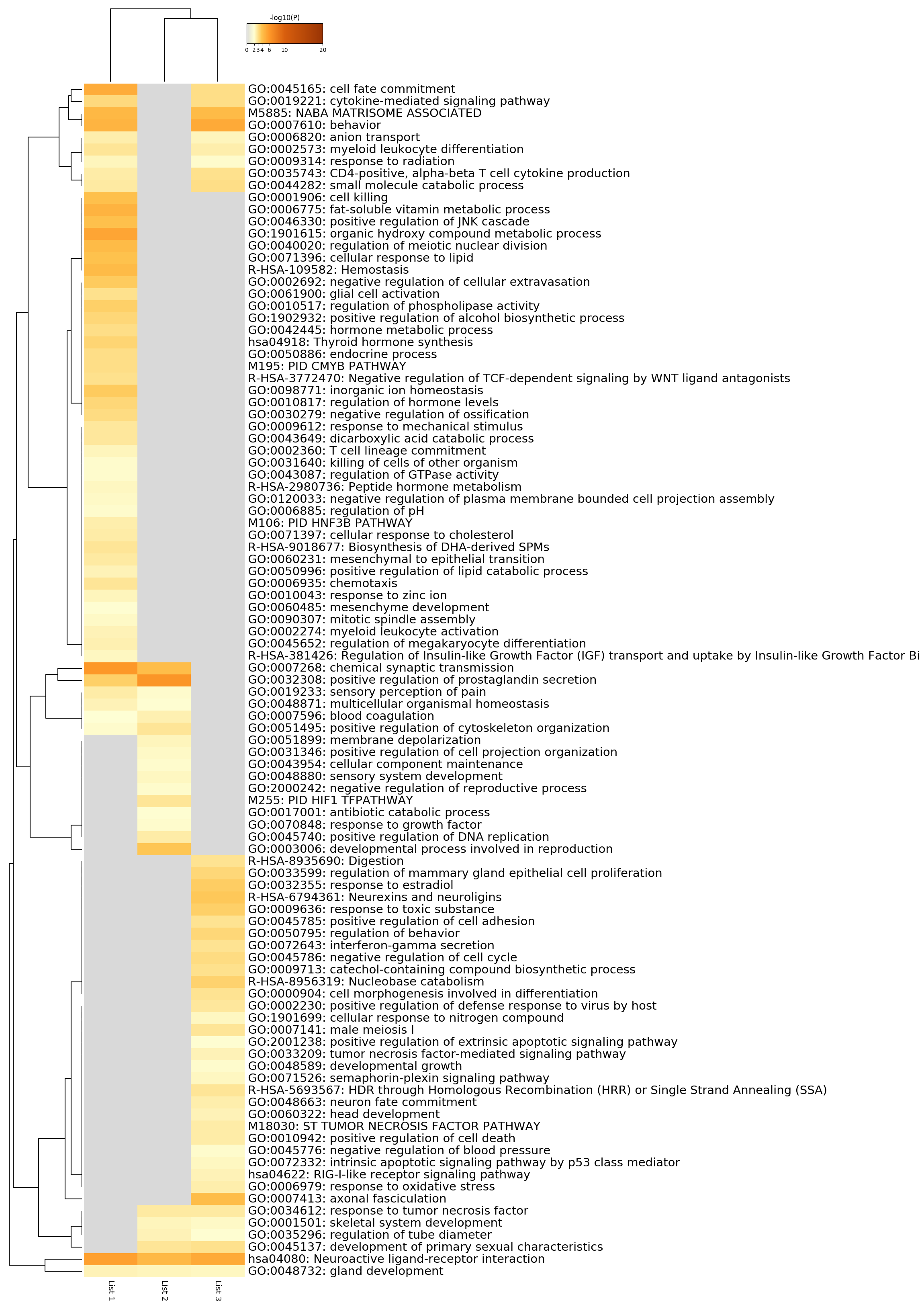

Supplement: Supplementary file 9 [file Image1.PNG]
